# Unique 40-year survival after heart transplantation with normal graft function and spontaneous operational tolerance

**DOI:** 10.1007/s00392-023-02341-x

**Published:** 2023-11-20

**Authors:** Wolfgang von Scheidt, Bruno Reichart, Bruno Meiser, Moritz von Scheidt, Partho Sen, Florian Schwarz, Eva Harmel, Frank M. Bengel, Andrea Dick, Peter Ueberfuhr, Hermann Reichenspurner, Elmar Jaeckel, Reinhard Schwinzer, Christian Hagl

**Affiliations:** 1 I.Medizinische Klinik, University Hospital Augsburg, University of Augsburg, Stenglinstr. 2, 86156 Augsburg, Germany; 2 Ludwig-Maximilians-University Munich, Munich, Germany; 3 Department of Cardiac Surgery, University Hospital Großhadern, Ludwig-Maximilians-University Munich, Munich, Germany; 4 Transplant Center, University Hospital Großhadern, Ludwig-Maximilians-University Munich, Munich, Germany; 5 Department of Cardiovascular Diseases, German Heart Center Munich, Technical University Munich, Munich, Germany; 6 Department of Diagnostic and Interventional Radiology, University Hospital Augsburg, University of Augsburg, Augsburg, Germany; 7 Department of Nuclear Medicine, Hannover Medical School, Hannover, Germany; 8 Laboratory for Immunogenetics and Molecular Diagnostics, University Hospital Großhadern, Ludwig-Maximilians-University Munich, Munich, Germany; 9 Department of Cardiovascular Surgery, University Heart and Vascular Center Hamburg, Hamburg, Germany; 10 Ajmera Transplant Center, UHN, University of Toronto, Toronto, Canada; 11 Department of General-, Visceral- and Transplantation-Surgery, Hannover Medical School, Hannover, Germany; 12 DZHK (German Centre for Cardiovascular Research), Partner Site Munich Heart Alliance, Munich, Germany

**Keywords:** Heart transplantation, Long-term survival, Immunosuppression, Tolerance, Myocardial function, Cardiac allograft vasculopathy

## Abstract

Unique 40-year survival after heart transplantation with normal graft function and spontaneous operational tolerance.

**Supplementary Information:**

The online version contains supplementary material available at 10.1007/s00392-023-02341-x.

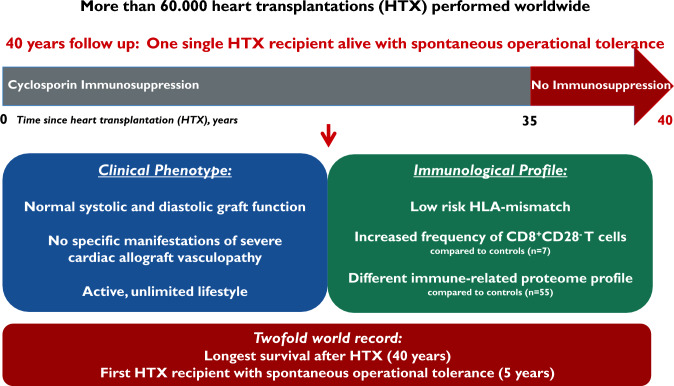

Sirs,

The introduction of cyclosporine in the early 1980s transformed heart transplantation from a desperate ultima ratio option into a convincing treatment for terminal heart failure [1]. In later years, newer immunosuppressive compounds expanded the success. Although the prognosis after heart transplantation is considerably better than the natural course of terminal heart failure, life expectancy remains limited [2, 3]. Long-term risks include cardiac allograft vasculopathy (CAV), graft failure, renal failure, infections and malignancies [1]. Most of these are either related to side effects of immunosuppression or to insufficiently controlled alloimmunity. Longevity, i.e., normalization of life expectancy, preferentially based on alloimmune graft acceptance, remains the most important, but unrealized ambition.

ECG, transthoracic echocardiography (EPIQ Diagnostic Ultrasound System CVx Release 7.0, Philips, Amsterdam, The Netherlands and TOMTEC Imaging Systems, Unterschleissheim, Germany), cardiac catheterization including coronary angiography (Artis Zee, Siemens, Erlangen, Germany), and cardiac MRI (Aera 1.5 Tesla, Siemens, Erlangen, Germany) were performed according to normal standards of a high-volume tertiary university hospital.

The recipient was actually HLA-11 loci typed (HLA-A, B, C, DRB1, DRB345, DQA1, DQB1, DPA1 and DPB1) on intermediate resolution level (LABType™SSO Typing Kits, One Lambda). Since the donor was typed in 1983 typing information for HLA-C and -DQ are lacking, the most likely donor HLA-C and -DQ antigens were assumed based on the known HLA-B and -C and -DR and -DQ-linked associations. The presence of de novo HLA- antibodies was tested using Luminex based bead assay technique (LABScreen™ mixed Class I&II and LABScreen™ Single Antigen HLA Class I and Class II, One Lambda). For molecular matching, HLAMatchmaker™ was used to calculate type and number of eplet mismatches between donor and recipient (eplet = functional part of the epitope). With the Epitope Registry, eplets can be classified according to their immunogenicity from very low to high immunogenic. The PIRCHE II SOT module helps to determine the total amount of foreign peptides presented by self-HLA class II molecules (PIRCHE: Predicted Indirectly Recognizable HLA Epitope).

Peripheral blood mononuclear cells (PBMC) were isolated by Ficoll-density gradient centrifugation. Control cells (7 healthy blood donors) were obtained from the cell bank of the Dept. of General-, Visceral-, and Transplantation Surgery or from anonymized/coded Leukotrap filters. Samples from elderly blood donors (mean age 68.8 ± 5.5 years) were used to correspond to the age of the patient (83 years). The following antibodies were purchased from BD Biosciences, San Jose, CA: CD3 (UCHT1), CD4 (RPA-T4), CD8 (RPA-T8), CD25 (M-A251), Foxp3 (259D/C7), CD19 (HIB19), and CD56 (B159). CD38 (HB-7) and CD24 (ML5) were from BioLegend, San Diego, CA. Stained cells were analyzed on a FACSCalibur flow cytometer (Becton Dickinson, San Jose, CA) and data were processed using FCS Express 7 (De Novo software, Pasadena, CA). In vitro proliferation assays were performed using RPMI 1640 culture medium supplemented with 10% FCS, 50 U/mL penicillin, 4 mM L glutamine, 50 µg/mL streptomycin, 1 mM sodium pyruvate, and 0.05 mM ß-mercaptoethanol.

The section on multiomics profiling from blood and PBMCs can be found in the Supplementary Information.

On May 18th, 1983, a 43-year-old male patient with end-stage dilated cardiomyopathy underwent orthotopic heart transplantation at the University of Munich Hospital Center. The donor, a 19-year-old male accident victim, had blood type O, the recipient B. Since the donor was in the same hospital as the recipient, ischemic time could be limited to less than 90 min. Transplantation was performed using biatrial, aortic and pulmonary artery anastomoses. Cyclosporine was administered immediately before and after the operation (17 mg/kg on day 1). 500 mg methylprednisolone was given i.v. after restoration of spontaneous circulation. No induction therapy was given. High-dose cyclosporine (12–15 mg/kg/day in two divided doses) was given in the first month, then tapered gradually (7–10 mg/kg/day until month 6, 3–5 mg/kg/day until year 5, and 2–2.5 mg/kg/day thereafter to a targeted trough level range of 100–150 ng/ml). Prednisone was tapered to 5 mg twice daily 2 months after heart transplantation, 5 mg daily at year 5, and stopped in 1992. Eight mild cellular rejections occurred in the first 8 months, which at that time (7 years before publication of a first treatment consensus) were treated each with 1 g methylprednisolone i.v. for 3 days. Moderate or severe rejection episodes (≥ grade 2 or 2R) were never observed. Azathioprine (25 or 50 mg daily) was added during years 6–11. In 1994, the patient stopped azathioprine arbitrarily. In 2018, he also stopped cyclosporine mono-therapy based on personal choice because of concerns that actinic keratosis of his head may progress to squamous cell carcinoma.

Systolic and diastolic myocardial function remained normal over the next 40 years, with an ejection fraction consistently exceeding 60%. All ECG’s documented sinus rhythm. In 1997, eccentric intimal proliferation with positive remodeling, as well as epicardial and microvascular endothelial dysfunction were documented by intravascular ultrasound and Doppler flow measurement. Angiographically, mild stenotic coronary lesions were observed for the first time in 2008. In 2015, a severe focal stenosis of the mid left anterior descending artery (LAD) was treated with drug-eluting stent implantation, but a diffuse CAV pattern was absent. ^11^C-hydroxyephedrine PET, performed 15 years after heart transplantation, documented persistent complete cardiac denervation. Over 40 years, the patient never experienced any severe infection requiring hospitalization.

The patient operated his own practice as a psychotherapist until he was 68 years old. He traveled worldwide without any restrictions. Today, he still maintains an active lifestyle. He denies any cardiopulmonary symptoms or limitations.

Present medication consists of apixaban 5 mg twice daily (due to deep vein thrombosis in 2020), ramipril 10 mg daily, and, irregularly, simvastatin 20 mg daily. More effective statins (atorvastatin, rosuvastatin) were not tolerated due to myalgias. Aspirin 100 mg daily was given 1993–2020, Clopidogrel 75 mg daily for 6 months after PCI in 2015.

*Physical examination* (180 cm, 91 kg, BMI 28.1 kg/m^2^, BP 110/60 mmHg, normal auscultation) and *ECG* (sinus rhythm, 94 bpm, normal time intervals, ST-segments and T waves) are unremarkable. *Laboratory studies* demonstrate slightly elevated NT-proBNP of 1004 pg/ml (ULN < 486), hs-troponin T of 22 pg/ml (ULN < 15), total cholesterol of 245 mg/dl, and LDL-C of 143 mg/dl (no statin intake in the last 2 months). Creatinine 1.09 mg/dl, GFR > 60 ml/min × 1.73 m^2^, HDL-C 79 mg/dl, triglycerides, glucose, HbA1c, and hemoglobin are in normal range.

*Transthoracic echocardiography* (video 1 and 2) reveals a normal enddiastolic diameter of the left ventricle (53 mm), a normal left ventricular ejection fraction (LVEF) of 65%, and a normal peak systolic global longitudinal strain of − 23%. E/A mitral flow ratio is 1.2. A normal E/e’ ratio of 8 is compatible with a non-elevated left ventricular enddiastolic pressure (LVEDP). Early-diastolic lateral mitral ring tissue Doppler velocity is normal (12 cm/s), making a restrictive diastolic dysfunction unlikely. Aortic and mitral valve leaflets are inconspicuous, no mitral regurgitation is seen. The combined donor-recipient left (56 ml/m^2^) and right atrium (54 ml/m^2^) are severely enlarged. The right ventricle is not dilated (28 mm), systolic contraction is normal (TAPSE 22 mm). Minimal tricuspid regurgitation is present. Right ventricular systolic pressure amounts to 30 mmHg plus central venous pressure. *Exercise stress echocardiography* reveals a normal increase of contraction in all segments up to 75 W. The maximum heart rate is 148 bpm after 9 min of exercise.

*24 h-Holter-ECG* shows regular sinus rhythm, mean heart rate is 94 bpm (range 68–121 bpm). Mean heart rate at daytime is 102 bpm, during night 86 bpm. Heart rate variability is markedly reduced: SDNN 54 ms, SDANN 47 ms. Very rare monotopic ventricular (155/24 h) and supraventricular extrasystoles (95/24 h) are present.

*Cardiac MRI* (Fig. 1, videos 3 and 4) shows a normal-sized left and right ventricle (enddiastolic volume index 50 and 45 ml/m^2^, respectively) with homogeneous, normal contraction (LVEF 62%, RVEF 48%). The calculated cardiac output is 5.5 l/min., and the left ventricular mass index is normal (71 g/m^2^). T2/T1 mapping and T2 STIR sequences exclude myocardial edema or diffuse fibrosis. Myocardial perfusion at rest is normal in all segments. Late enhancement sequences reveal a unifocal area of gadolinium enhancement in the basal septum, consistent with the typical target region for obtaining endomyocardial biopsies (Figure S1). No mitral or tricuspid regurgitation jets are seen. The left and right atrium are severely enlarged (44 and 39 cm^2^, respectively).

**Fig. 1:**
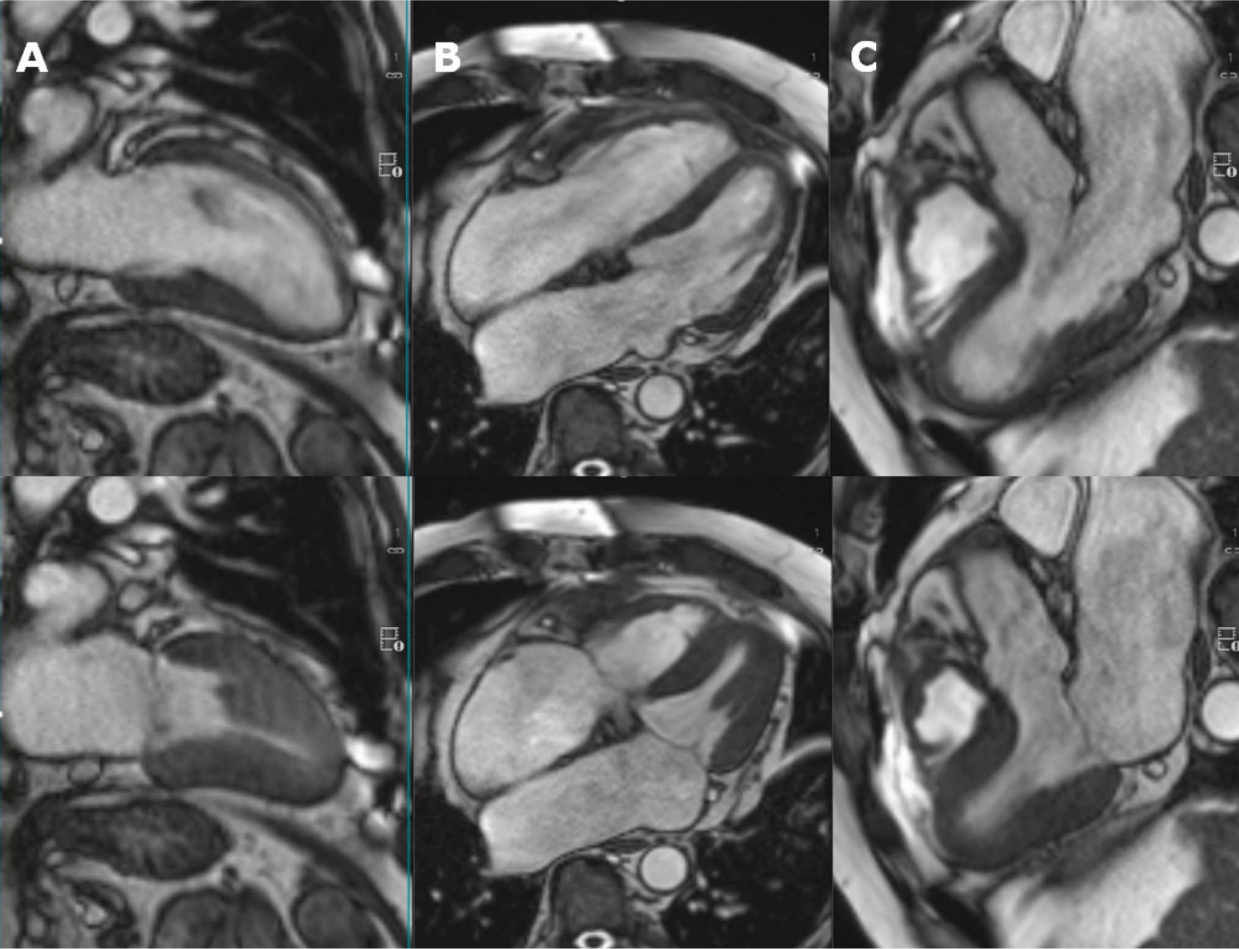
Cardiac MRI. Upper panel diastole, lower panel systole. **A** 2-chamber view. **B** 4-chamber view. **C** 3-chamber view. See also video 2A and B. A homogeneous, normal left ventricular contraction is seen, ejection fraction amounts to 62%. Left ventricular mass index is normal (71 g/m^2^). Combined recipient-donor atria are severely enlarged

The most recent *cardiac catheterization* (June 2021) reveals a LVEDP in the upper normal range (15 mmHg), a normal LVEF of 70%, and no mitral regurgitation (Fig. 2). Coronary angiography is shown in Fig. 2.

**Fig. 2:**
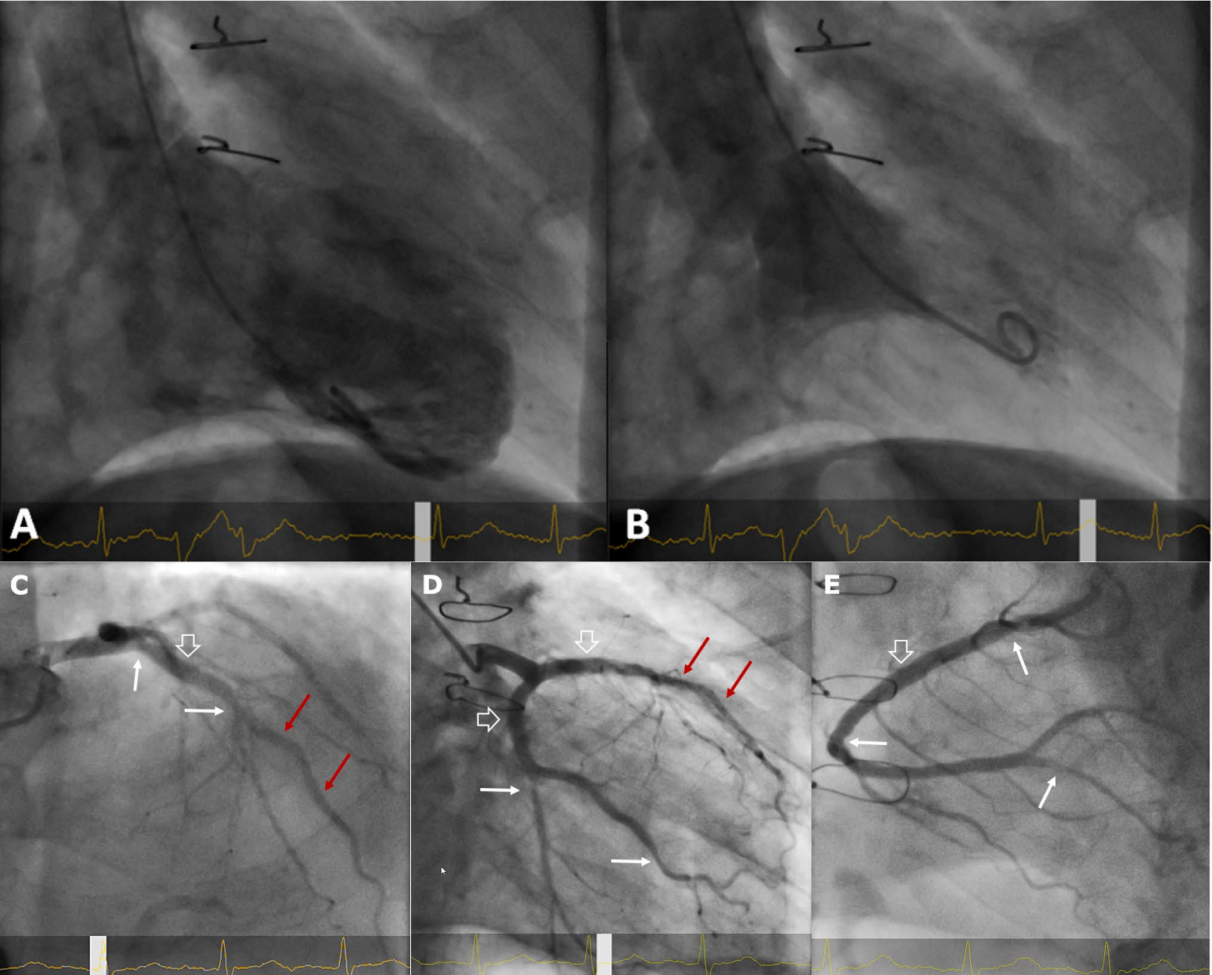
Left ventriculography and coronary angiography. **A**, **B** Left ventriculography (A diastole, B systole, 30° RAO view), June 2021, three years after cessation of immunosuppression. Normal ejection fraction of 70%. **C**–**E)** Coronary angiography, June 2021. **C** left coronary artery, RAO cranial view. A focal 30% ostial LAD and 40–50% mid-LAD narrowing is seen (white arrows), in between a mild dilated angiopathy of the proximal LAD (open white arrow). Stent of the mid-LAD without restenosis (red arrows). Ostial, small diagonal branch severely narrowed, 40% narrowing of the septal branch. **D** left coronary artery, RAO caudal view. 50% narrowing of the mid-circumflex artery and the marginal branch (white arrows), mild dilated angiopathy of proximal circumflex artery and LAD (open white arrows). Stent of the mid-LAD unremarkable (red arrows). **E** Right coronary artery, AP cranial view. Focal 30% proximal and 40% mid-segment narrowing (white arrows), in between a mild dilated angiopathy of the proximal segment (open white arrow). Proximal 70–80% stenosis of the posterior descending artery (white arrow), with subsequent irregularities. Note that significant rarefication of side branches or diffuse tapering of distal vessel segments as specific manifestations of CAV are absent

The *HLA* phenotypes of the recipient and donor are HLA-A1,68; B8,44; Cw7; DR17,11; DQ2,7 and HLA-A3,24; B62,39; DR1,5, respectively. HLA-Cw7, Cw9, DQ5, and DQ7 are assumed as the most likely donor HLA-C and -DQ antigens based on the known HLA-B and -C and -DR and -DQ-linked associations. The patient did not develop any donor-specific anti-HLA class I and class II antibodies. Using published calculations to estimate the risk of developing donor-specific de novo HLA-DQ antibodies, the number of mismatches in the patient is below the threshold for increased risk of alloimmunization, and known highly immunogenic eplet mismatches are not present [4].

Investigation of *lymphocyte subpopulations* reveals that neither CD4^+^ T cells nor B or NK cell populations are expanded in the patient compared to control samples from 7 healthy blood donors (mean age 68.8 ± 5.5 years, Table 1). However, there is a marked increase of CD3^+^CD8^+^ T cells (33.9% vs 13.0 ± 5%). Further characterization of the CD3^+^CD8^+^ population reveals a higher frequency of CD8^+^CD28^−^ T cells in the patient compared to controls (30% vs 5%, Fig. 3A). However, the high frequency of CD8^+^ T cells, expressing a regulatory phenotype, is not accompanied by reduced in vitro T-cell reactivity against alloantigen, as no differences between patient and control cells are found (Fig. 3B).

**Table 1:** Frequencies of T, B, and NK subpopulations in lymphocytes from control individuals and the heart-grafted patient^a^

	T cells (CD3^+^)			B cells (CD19^+^)			NK cells
	CD4^+^	CD4^+^CD25^high^Foxp3^+^	CD8^+^		CD24^high^	CD38^+^	CD3^−^CD56^+^
Controls (*n* = 7)							
Mean	35.9^b^	1.8	13.0	10.9	2.1	8.7	26.1
SD^c^	14.0	1.0	5.0	4.6	1.0	3.1	18.2
Patient	34.6	1.0	33.9	3.6	0.7	3.2	4.5

^a^PBMC were isolated by Ficoll-gradient centrifugation, stained with appropriate antibody combinations and analyzed on a FACSCalibur flow cytometer

^b^Results are expressed as % positive cells among “gated” lymphocytes. For controls, mean values and standard deviations are given as obtained by the analysis of samples from 7 healthy individuals (mean age 68.8 ± 5.5 years). Patient data represent mean values obtained from quadruple measurements

^c^Standard deviation

**Fig. 3:**
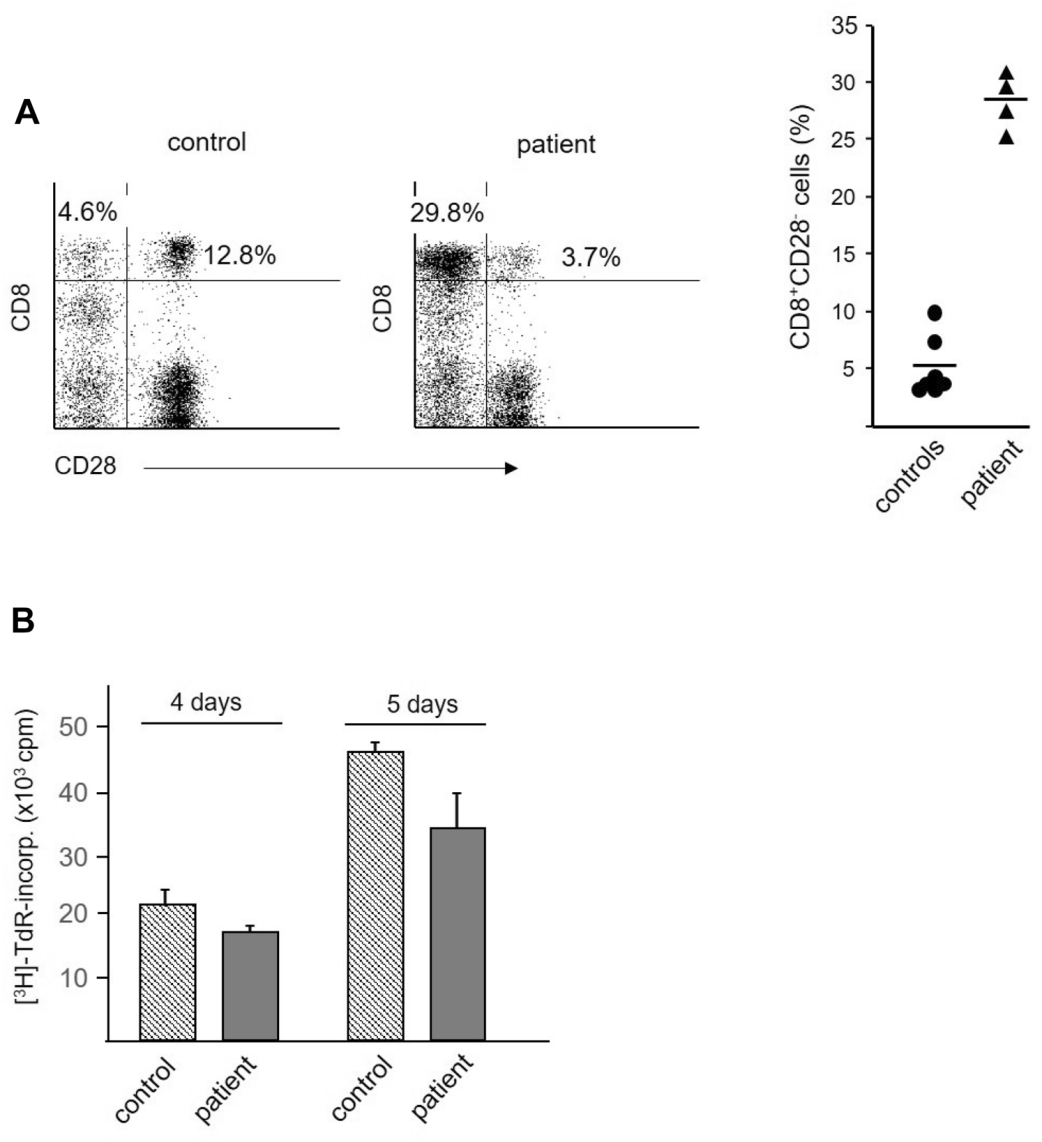
Immunologic characterization of cells. Immunologic characterization of cells from healthy controls (mean age 68.8 ± 5.5 years) and the heart-grafted patient. Peripheral blood mononuclear cells (PBMC) were isolated by Ficoll-gradient centrifugation and used for phenotypic and functional studies. **A** Enhanced frequency of CD8^+^CD28^−^ T cells in patient’s blood samples. Cells were stained with the antibody combination CD3/CD8/CD28 and analyzed on a FACSCalibur flow cytometer. Analyses were performed on “gated” lymphocytes as defined by forward- and side-scatter characteristics. *Left panels,* representative dot-plots of CD8/CD28 co-expression patterns. Numbers represent percentage of bright CD8^+^ (CD3^+^) T cells lacking CD28 expression (upper left quadrant) or co-expressing CD28 (upper right quadrant). *Right panel,* summary of studies using samples from 7 healthy control individuals and quadruple analyses of the blood sample obtained from the patient. The horizontal markers represent mean values of each group. **B** Similar intensity of alloantigen-induced proliferation in control and patient cells. Responder PBMCs (1 × 10^5^/well) were stimulated with irradiated (30 Gy) allogeneic cells (1 × 10^5^/well, pooled PBMC from five different blood donors). The cells were cultured for 4 and 5 days, pulsed with tritiated thymidine and harvested after 16 h. One representative experiment is shown, similar data were obtained in a second experiment. Results are expressed as mean counts per minute (cpm) ± SD of triplicate cultures

*Transcriptome analysis* of the patient identifies a total of 226 and 288 highly abundant genes in the PBMCs and blood samples, respectively. The co-expression analysis of these genes reveals functional modules related to T-cell-mediated immune processes and the formation of MHC-protein complexes. Interestingly, some genes such as *HLA-A and E*, *VSIR*, and *NCKAP1L* have been found to be associated with CD8^+^ alpha–beta T-cell activation (Fig. 4A and D). In addition, the fold enrichment analysis identifies gene ontology (GO) terms associated with the regulation of immune responses, MHC class I complex, T-cell activation, T-cell-mediated cytotoxicity, and cytokine production (Fig. 4B). T-cell-mediated immune processes are significantly overrepresented based on PBMC data (Fig. 4C). In summary, transcriptomic analysis identifies gene modules associated with activation and regulation of T-cell-mediated immunity. *Mass spectrometry (MS)-based proteomics* identifies 12 highly abundant proteins from the patient's serum samples, primarily associated with immune processes, and lipid metabolism (Fig. 5A). Co-expression analysis of these abundant proteins reveals several functional modules, including immune response, lipid biosynthesis, steroid esterification, cholesterol transport, and lipid particle remodeling (Fig. 5A). Most of these proteins were synthesized by the *APOA* and *APOE* genes. In summary, serum proteins are predominantly linked to immune processes and lipid metabolism.

**Fig. 4:**
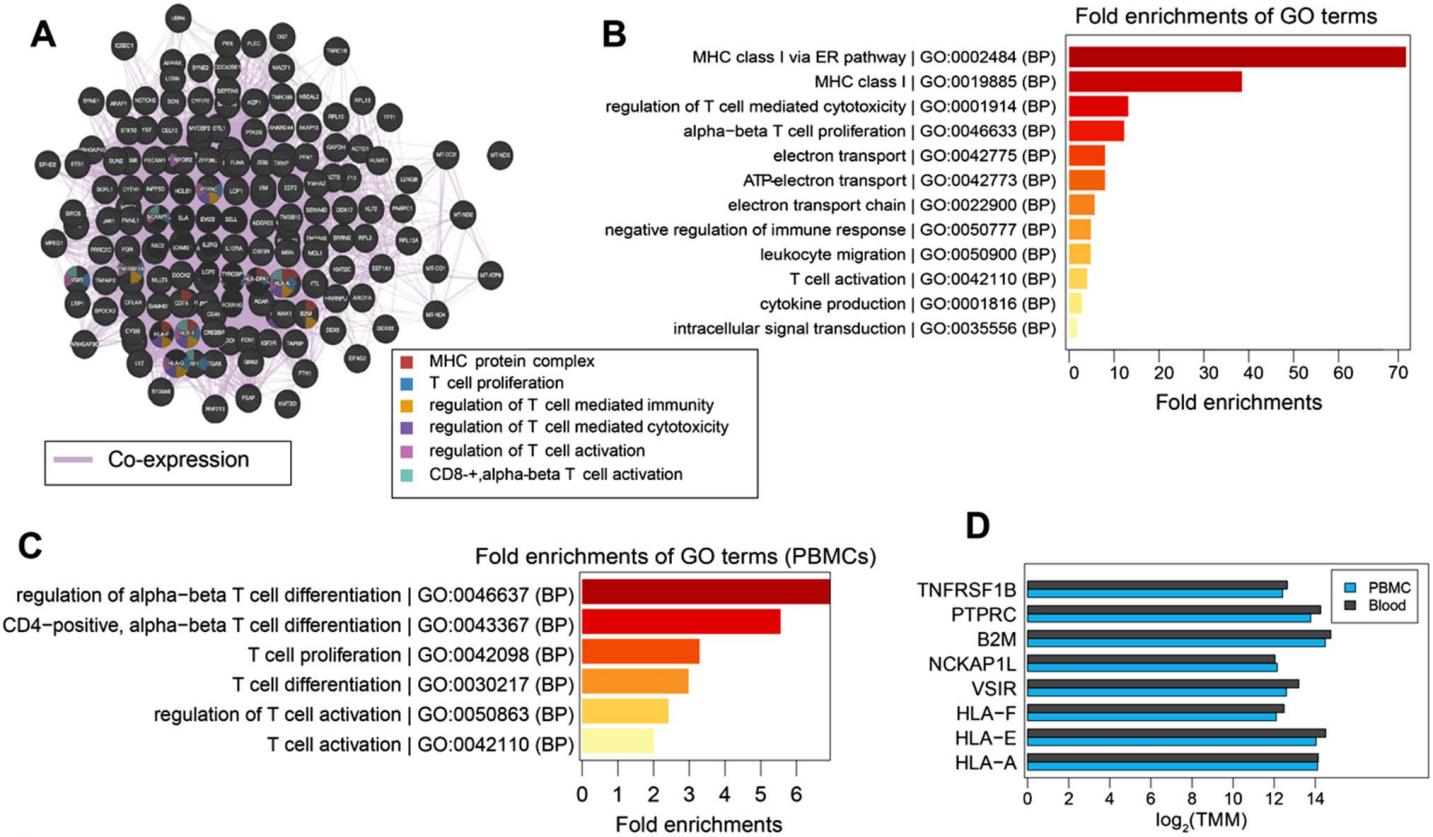
Transcriptomic profiling. Transcriptomic profiling and comparison of gene modules associated with T-cell-mediated processes in PBMCs and blood samples of the patient. **A** Provided is a co-expression graph where each node represents a gene and edges represent co-expression scores, with thicker edges indicating stronger correlations between genes. Genes shared by different modules are color-coded according to their function. **B** Displays the fold enrichment of the GO terms associated with T-cell-mediated processes derived from the combined list of abundant genes (transcriptomics) in PBMCs and blood samples of the patient (*n* = 1). **C** The fold enrichments of the GO terms associated with T-cell-mediated processes derived from the list of abundant genes (transcriptomics, *n* = 1) represented in the PBMCs. **D** The expression of genes associated with T-cell-mediated processes in PBMCs and blood samples of the patient (*n* = 1), represented by the log_2_ weighted trimmed mean of M-values (TMM). In summary (**A**–**D**), T-cell-mediated immune response, and MHC class I pathways are differentially regulated. *B2M*: beta-2-microglobulin, *HLA-A*: HLA class I histocompatibility antigen A alpha chain, *HLA-E*: HLA class I histocompatibility antigen alpha chain E, *HLA-F*: HLA class I histocompatibility antigen alpha chain F, *NCKAP1L*: nck-associated protein 1-like, *PTPRC*: receptor-type tyrosine-protein phosphatase C, *TNFRSF1B*: tumor necrosis factor receptor superfamily member 1B, *VSIR*: v-type immunoglobulin domain-containing suppressor of T-cell activation

**Fig. 5:**
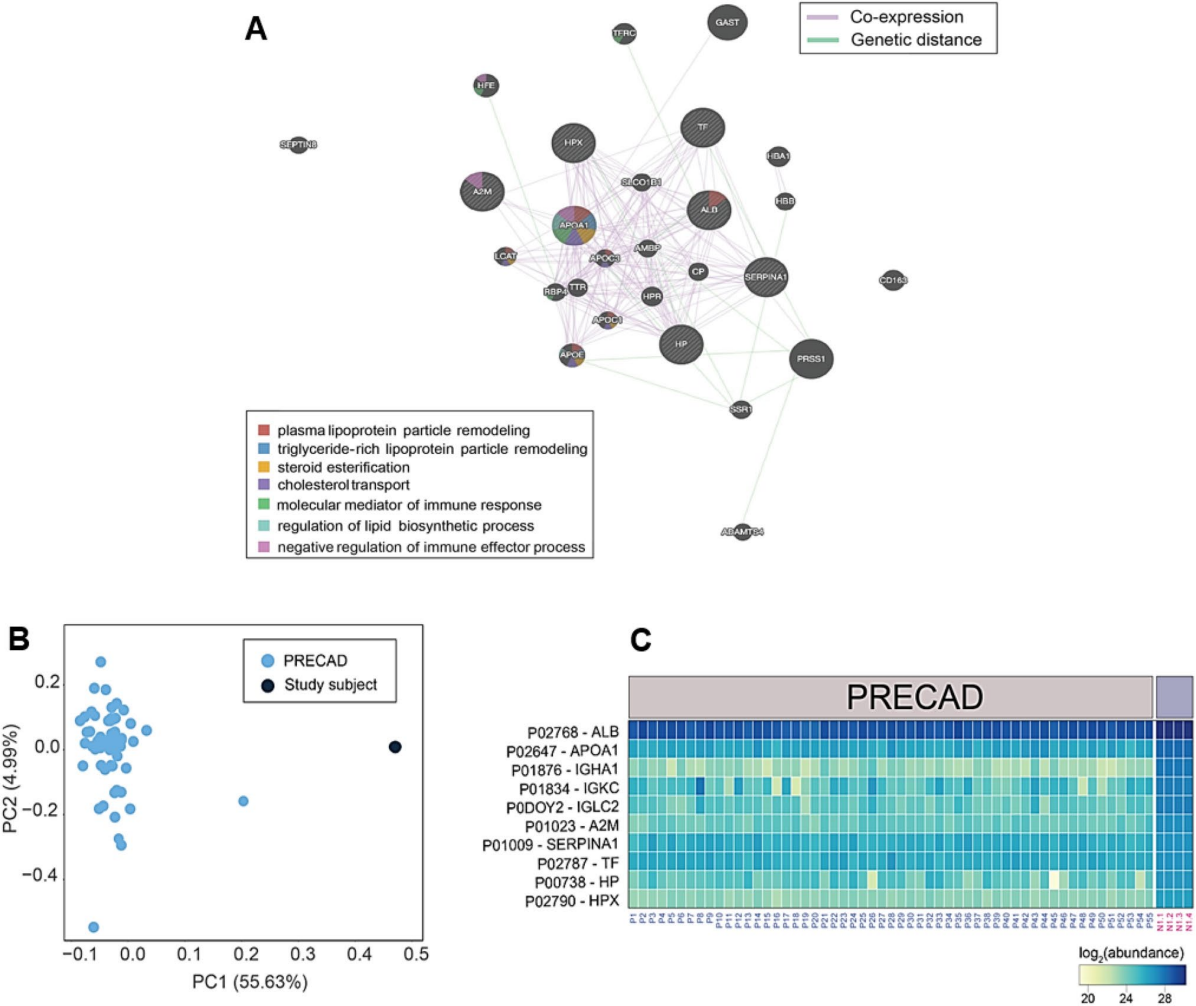
Proteomic profiling. **A** Provided are information about the protein modules associated with immune regulation, lipid metabolism, and steroid biosynthesis in the blood samples of the patient. Shown is a co-expression graph where each node represents a gene and edges represent co-expression scores, with thicker edges indicating stronger correlations between genes. Genetic distances between genes within a module are indicated by green lines. Genes shared by different modules are color-coded according to their function. In summary, immune regulatory processes, and lipid metabolism are most differentially regulated. **B** A score plot that depicts a principal component analysis (PCA) performed on the proteome of 55 individuals with CAD involved in the PRECAD study, as well as the patient (study subject, *n* = 1). **C** Heatmap of the log_2_ abundances of the most abundant proteins in the patient (*n* = 1, four replicated denoted by N1.1–4), compared with the protein abundances of 55 matched individuals with CAD who participated in the PRECAD study. Protein identifiers are followed by their corresponding entrez gene names. In summary, clear differences are most significant regarding immune system-related traits. *A2M* alpha-2-macroglobulin, *ALB* albumin, *APOA1* apolipoprotein A-I, *HP* haptoblobin, *HPX* hemopexin, *IGHA1* immunoglobulin heavy constant alpha 1, *IGKC* immunoglobulin kappa constant, *IGLC2* immunoglobulin lambda constant 2, *SERPINA1* alpha-1-antitrypsin, *TF* serotransferrin

Compared to age (83.0 vs 82.7 ± 1.6 years) and coronary artery disease complexity (GENSINI score 63.0 vs 63.0)-matched individuals (*n* = 55) from the PRECAD study, 145 (out of 300) overlapping proteins are identified in our patient. The proteomic signature differs significantly as compared to controls (Fig. 5B). Interestingly, most of these proteins (*A2M*, *ALB*, *APOA1*, *HP*, *HPX*, *IGHA1*, *IGKC*, *IGLC2*, *SERPINA1* and *TF*) show higher abundances in the patient (Fig. 5C). In summary, clear differences are most significant regarding immune system-related traits.

The median survival after heart transplantation performed in the years 1980–1985 and 2002–2009 was 5.3 and 12.5 years, respectively [2]. Very long-term survival amounts to less than 1% after 30 years [3]. The life expectancy of a 43-year-old German male in 1983 was 74 years [5]. Thus, the now 83-year-old patient has gained the maximum achievable benefit, i.e., heart transplantation normalized his former dismal prognosis.

In the early cyclosporine era, high doses of cyclosporine and longer term use of prednisone were common. In the late 80 s, triple immunosuppression (including azathioprine) gradually became standard [6]. Although the patient reduced his immunosuppression by personal choice to cyclosporine mono-therapy in 1994, and stopped cyclosporine completely in 2018, relevant rejections never occurred. Monotherapy using the calcineurin inhibitor tacrolimus has been performed safely in selected patients [1, 7, 8]. Significant intimal proliferation and endothelial dysfunction are known predictors of a worse prognosis [9, 10]. However, a minority of patients exhibits signs of adaptive, positive vascular remodeling, thus preventing or delaying vessel and lumen shrinkage [1, 11–13]. Although a single PCI-requiring focal stenosis formally indicates moderate CAV according to the ISHLT classification, specific angiographic manifestations of CAV, e.g., diffuse distal pruning, abrupt distal occlusions or side branch rarefication, were never seen in our patient [14]. Thus, conventional coronary atherosclerosis may be the leading manifestation in the present case.

The absence of severe CAV and relevant cellular rejections may explain the preserved systolic and diastolic myocardial function over 40 years. Of note, patchy or diffuse, ischemic or non-ischemic fibrosis or scarring, not uncommon late after heart transplantation, is completely absent on cardiac MRI. Focal septobasal fibrosis most probably is a consequence of repeated biopsies. Even late after heart transplantation, borderline elevations of hs-troponin T are a normal finding without clinical significance [15]. The unusual electrical and mechanical quiescence of the recipient atrial remnants (once in permanent atrial fibrillation before transplantation) enables a bidirectional volume shift during contraction of the smaller donor atrial parts to the ventricles and to the recipient atrial remnants. This smaller atrial contribution to ventricular filling and the giant atrial geometry may explain the slightly elevated NT-proBNP values (≤ 2xULN), which are stable over the past 20 years.

Reinnervation was excluded by ^11^C-hydroxyephedrine PET 15 years after transplantation, making a persistent long-term denervation extremely likely [16, 17]. This is supported by the present Holter ECG results. Although persistent lack of reinnervation is not associated with worse prognosis, consequences of denervation include lack of anginal warning symptoms in case of severe CAV and subnormal chronotropic and inotropic response to exercise [16–18]. Since immediate intracardiac neuronal norepinephrine release is blunted in denervated hearts, heart rate and contractility increase during exercise depend on circulating epinephrine and norepinephrine [19]. Remarkably, the peak exercise heart rate of 148 bpm in our patient, which is normal according to age, documents an unexpected adequate chronotropic exercise physiology.

Most of the historic long-term surveillance and treatment strategies are considerably simplified and modified today, e.g., no routine endomyocardial biopsies beyond year 1 in low-risk recipients, more selective use of coronary angiography, but including IVUS or OCT, implementation of noninvasive rejection- or CAV-diagnostics, cessation of corticosteroids 3–12 months after heart transplantation, tacrolimus and mycophenolate mofetil as standard immunosuppression, selective use of proliferation signal inhibitors beyond 6 months after transplantation in case of renal failure or progressive CAV [1, 8, 13, 20, 21].

Potential individual reasons for the pivotal feature, i.e., the exceptionally benign clinical course and host-graft-adaptation can be discussed. Several clinical predictors of longevity and preservation of myocardial function are present [1, 2]. The short ischemic time may be of special relevance.

The patient clinically exhibits operational tolerance of the transplanted heart for 5 years. The *HLA* mismatch of the recipient and donor in the present case is a low-risk constellation, and absence of donor-specific anti-HLA antibodies (DSA) seems to be relevant [4, 22–24]. However, neither the presence nor the absence of DSAs in the context of operational tolerance is predictive of long-term graft outcome [25].

Spontaneous immune tolerance has been described in some immunosuppression-free renal and, more frequently, liver transplant recipients [26, 27]. Spontaneous tolerance is associated with *regulatory/suppressive immune cells,* e.g., CD4^+^CD25^high^Foxp3^+^ and CD8^+^CD28^−^ regulatory T cells, and/or CD19^+^CD24^high^CD38^+^ B cells in peripheral blood, as has been found after renal transplantation [27–31]. However, graft-resident T-cell receptor repertoires may be different to those in the peripheral blood, as has been shown for liver allografts [32].

There is no known “tolerance signature” in heart transplant recipients. The expansion of the CD8^+^CD28^−^ T-cell population may indicate an involvement in tolerance development. CD8^+^CD28^−^ regulatory T cells have been described to maintain stable graft function after liver transplantation and they possess allospecific suppressive activity in vivo [33, 34]. In the CD8^+^CD28^−^ subset of heart-grafted patients, regulatory cells have been identified which might play a protective role for the graft [35].

Whatever cellular and molecular mechanisms are involved in the long-term acceptance of the patients’s heart, it is important that the immune tolerance seems to be graft-specific without systemic immune tolerance, as observed by the preserved allospecific peripheral immune response and the clinical absence of relevant infections. This might also be the major clinical benefit between unspecific immunosuppression and graft-specific immune control.

Increased frequencies of CD8^+^CD28^−^ T cells have also been described as biomarker of immunosenescence [36]. Since the control group is also of senior age (69 vs 83 years), immunosenescence is unlikely to be a relevant factor in our case. Nevertheless, even unspecific immunosenescence may attenuate the immune response [36].

Functional analysis of the *transcriptome and proteome* of the patient reveals specific alterations in immune processes. Several signatures associated with T-cell-mediated immune response, and MHC class I pathways are differentially regulated, suggesting that these biological processes may play a role in the patient’s operational tolerance and graft longevity. Although we do not have a control group for the transcriptome analysis, it is very unlikely that the selective overexpression of a T-cell pattern is a normal finding, given the broad range of immune system-related pathways. The proteomic comparison with age and coronary status matched individuals using principle component analysis visualizes clear differences, with immune system-related traits being the most significant factors at pathway level. Thus, downstream proteomic analyses confirm the transcriptomic findings and differ significantly from well-matched controls.

Of note, the data provided are primarily descriptive and give a first general impression of lymphocytic, transcriptomic and proteomic signatures, without causal relationship, an inevitable limitation of studying a single individual. It is clear that in a complex universe like the immune system, a specific or single immunological/molecular explanation for spontaneous tolerance cannot be expected. However, the notion that long-term operational tolerance is possible after heart transplantation is of major clinical importance. It may stimulate further clinical and experimental immunotolerance research and has important implications for long-term management of heart transplant recipients. Clearly, weaning of immunosuppression in selected heart transplant recipients should not be tested outside careful clinical trials, as emerging rejections may be immediately life-threatening. New tolerance inducing strategies are currently tested in kidney and liver transplantation (ClinicalTrials.gov Identifier: NCT04817774, NCT05234190), and this might become a potential future option even for heart transplant patients.

In summary, the phenotype presented here is exceptional in two aspects. First, the patient survived 40 years since heart transplantation and is presently, at the age of 83, still maintaining an active lifestyle, with normal myocardial function. Second, spontaneous operational allograft tolerance is present, a hitherto unreported phenomenon in clinical heart transplantation. This case documents that normal life expectancy and alloimmune graft acceptance, the ultimate goals, are possible after heart transplantation. Future interventions may realize tolerance induction in heart transplant recipients.

### Supplementary Information

Below is the link to the electronic supplementary material.

Supplementary file1 (DOCX 3508 KB)

Supplementary file2 (PDF 554 KB)

Supplementary file3 (MP4 6141 KB)

Supplementary file4 (MP4 4637 KB)

Supplementary file5 (MP4 22699 KB)

Supplementary file6 (MP4 29544 KB)

## Data Availability

The data underlying this article will be shared on reasonable request to the corresponding author.
