# Supplementary material for: Unique 40-year survival after heart transplantation with normal graft function and spontaneous operational tolerance
Source: Clin Res Cardiol. 2023 Nov 20;113(5):661–71. doi: 10.1007/s00392-023-02341-x (PMC11026283; doi:10.1007/s00392-023-02341-x)
Supplement: Supplementary file 1 — Supplementary file1 (DOCX 3508 KB) [file 392_2023_2341_MOESM1_ESM.docx]

**Supplementary Figure**


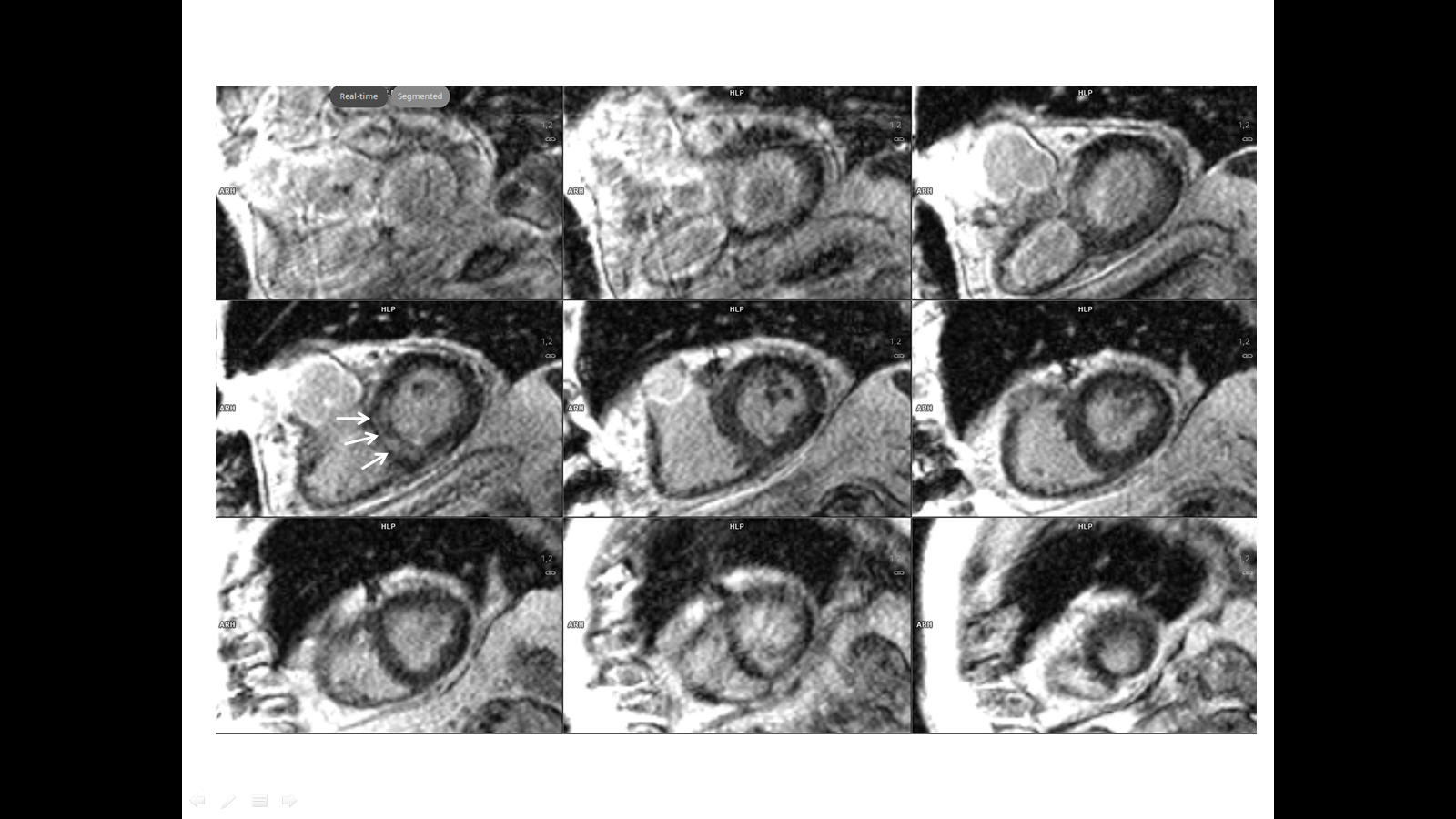


**Supplementary Figure S1. Cardiac MRI.** Late enhancement series, short axis view. Isolated, circumscript late enhancement, compatible with fibrosis, of the basal septum (arrows), the typical target region for endomyocardial biopsies.

**Video still images and Legends**

**
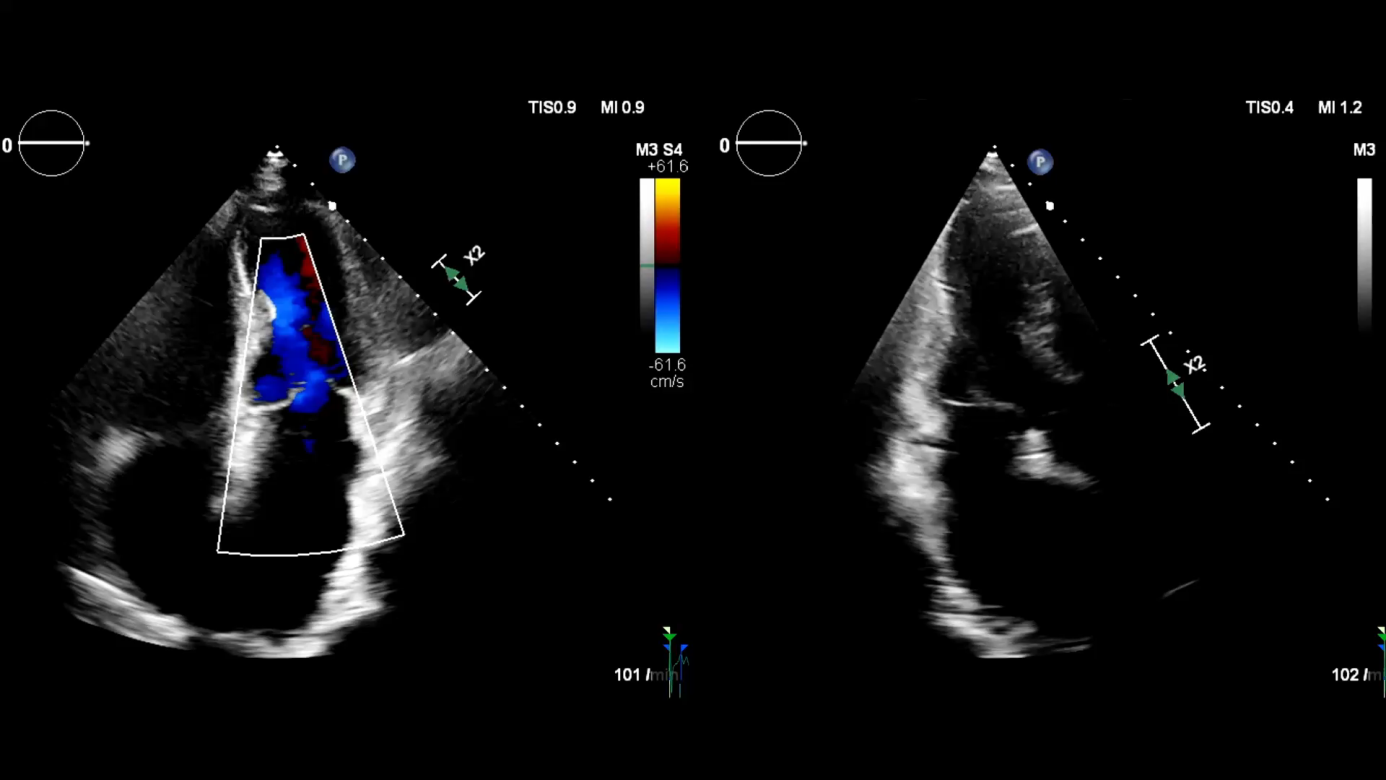
**

**Video 1. Transthoracic 2D-Echocardiography**. Color flow Doppler apical 4-chamber view (left) and 3-chamber view (right). A homogeneous left ventricular contraction and a normal ejection fraction of 65% is observed, no mitral regurgitation. Severely enlarged combined donor and recipient left and right atrium.

**
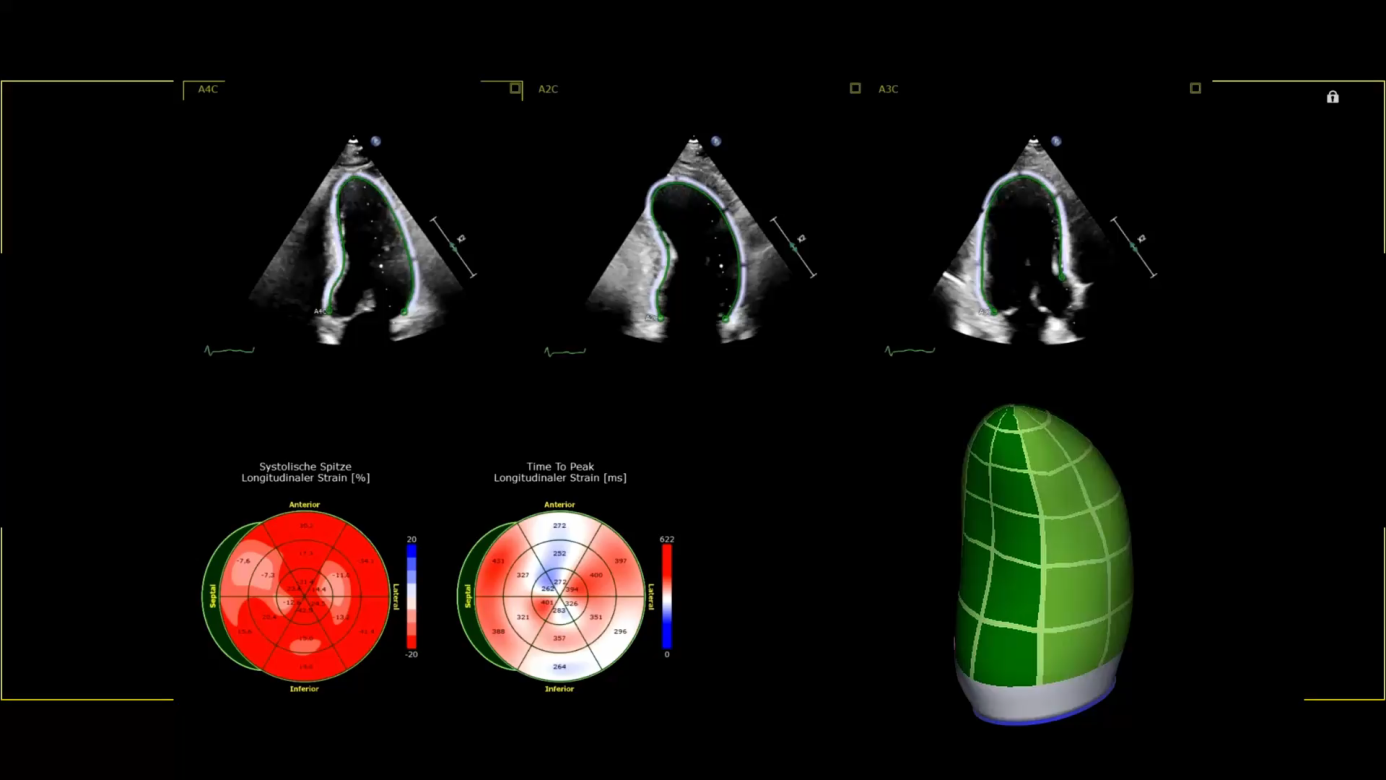
**

**Video 2. Transthoracic Echocardiography: Strain Analysis and 3D-Imaging.** Left ventricular apical 4-chamber view (left), 2-chamber view (middle) and 3-chamber view (right) showing endocardial tracing of speckle tracking echocardiography strain analysis (upper panel). Peak systolic global longitudinal strain is normal, amounting to -23% (bullseye analysis, lower panel, left). 3D-volume-imaging of left ventricle showing a normal, homogeneous contraction (lower panel, right)

**
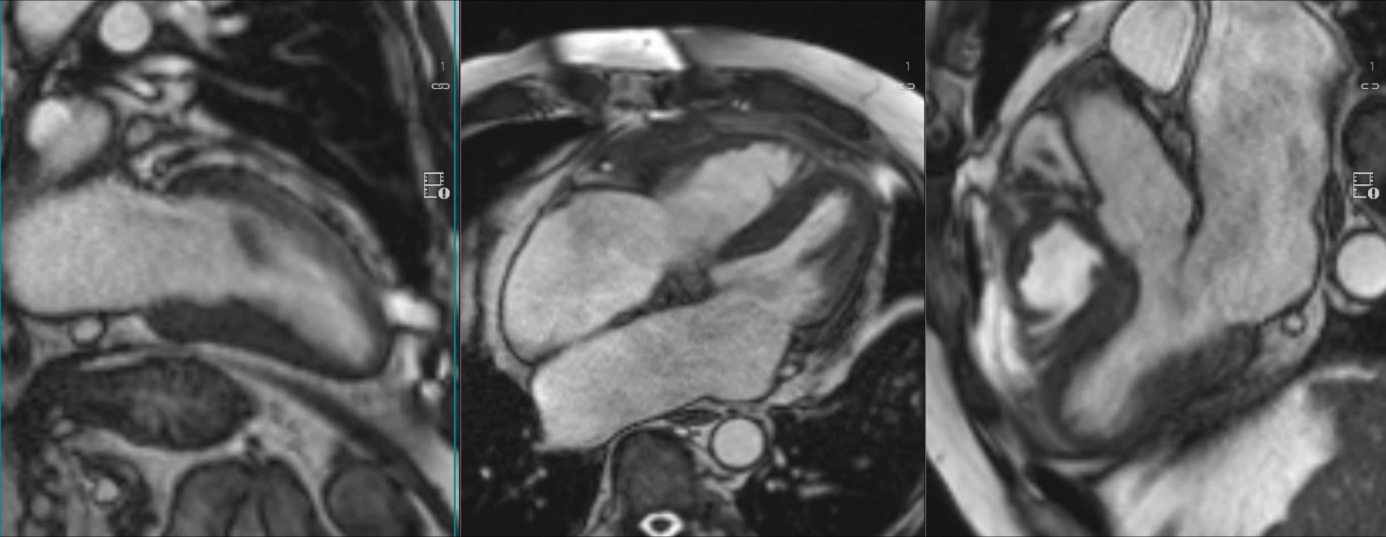
**

**Video 3. Cardiac MRI**. Left: 2-chamber view. Middle: 4-chamber view. Right: 3-chamber view. A normal, homogeneous contraction of both ventricles is seen, left ventricular EF amounts to 62%. Note the severely enlarged atria, best seen in the 4- and 3-chamber view, with atrial contraction in the donor part only, and non-contractile recipient remnants (posterior 2/3 of the atria). No mitral or tricuspid regurgitation is seen.

**
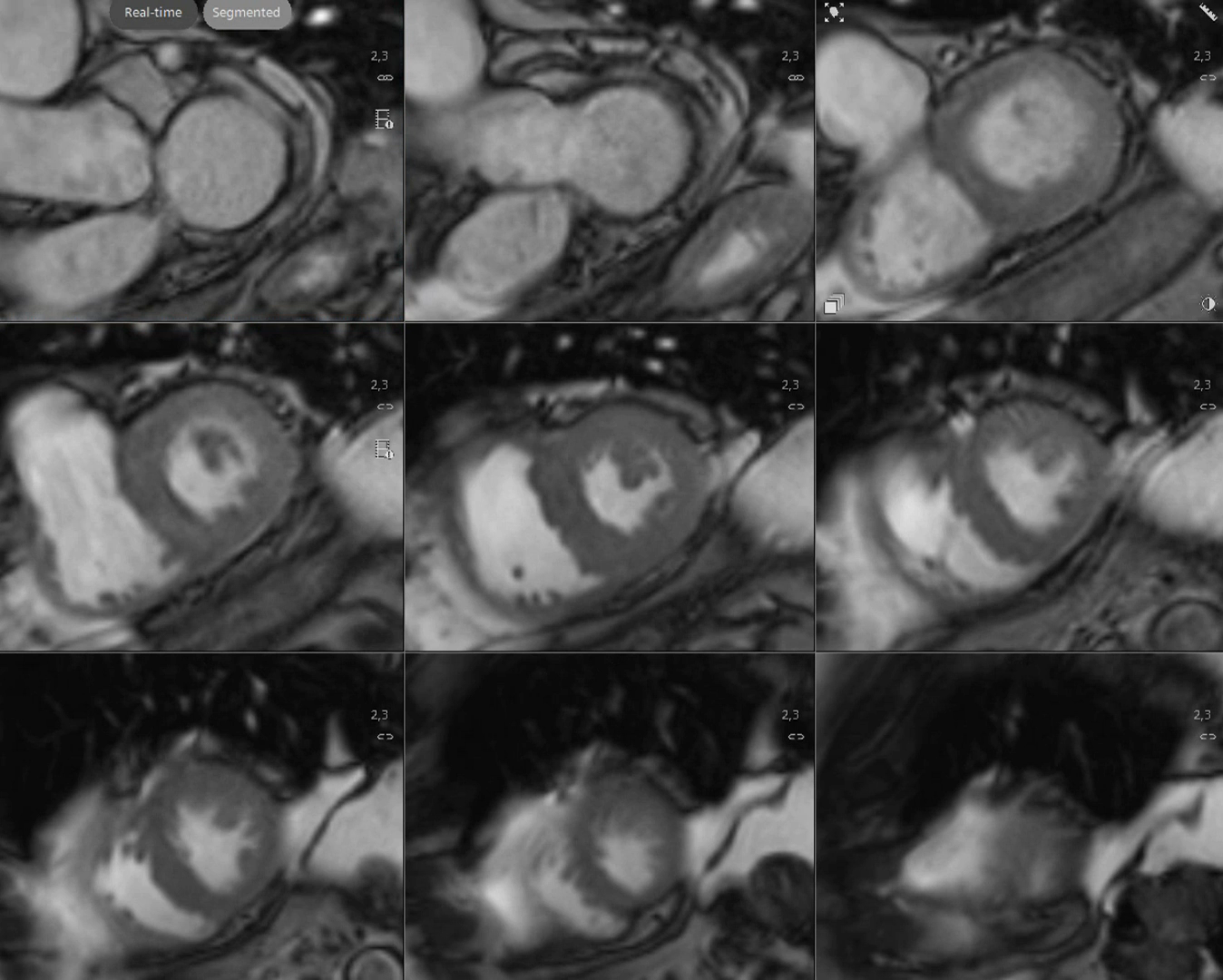
**

**Video 4. Cardiac MRI**. Short axis view of the left ventricle. A normal, homogeneous contraction is seen.
