# Supplementary material for: Unique 40-year survival after heart transplantation with normal graft function and spontaneous operational tolerance
Source: Clin Res Cardiol. 2023 Nov 20;113(5):661–71. doi: 10.1007/s00392-023-02341-x (PMC11026283; doi:10.1007/s00392-023-02341-x)
Supplement: Supplementary file 2 — Supplementary file2 (PDF 554 KB) [file 392_2023_2341_MOESM2_ESM.pdf]

Supplementary Information

Methods

Multiomics profiling from blood and PBMCs

Transcriptomics profiling and analysis. *Quality control, alignment, and data preprocessing.*

RNA sequencing (RNAseq) was performed on PBMCs (n=1) and PAX tube (n=1) blood samples obtained from the subject. RNA was extracted from PBMCs (Becton Dickinson, Switzerland) and whole blood (PAXgene, Becton Dickinson, Switzerland) without freezing step in a semi-automated process using a Maxwell RSC simplyRNA Tissue (Promega) kit on a Maxwell RSC48 (Promega) platform. All samples reached RNA integrity numbers (RIN) above 7 as initial quality control. Sequencing was performed at the Munich Leukemia Laboratory (MLL), Munich (NovaSeq™ 6000Dx System, Illumina).

The raw sequencing data (.fastq.gz) was subjected to quality control using FastQC (version=0.12.1) to assess various quality metrics, such as per-base sequence quality, per-sequence quality scores, GC content, and sequence length distribution [1]. Trimmomatic (version 0.39 was used with *"LEADING:5 TRAILING:5 SLIDINGWINDOW:5:10 MINLEN:50"* parameters to remove low-quality reads, adapter sequences, and contaminating sequences from the raw sequencing data [2]. The trimmed reads were aligned to the GRCh38 reference human genome using STAR software (version 2.7.10b_alpha_230301) [3]. The *"quantMode GeneCounts"* option in STAR was used to estimate the read counts. To account for differences in sequencing depth and library size, read counts were normalized using the TMM (weighted trimmed mean of M-values) method implemented in the "DESeq2 (version 1.30.1)" R package, which combines read count data from two different lanes of Illumina sequencing [4]. The resulting normalized read counts were used for downstream analysis.

Proteomic profiling and analysis. *Control group and sample preparation*. As part of the personalized risk prediction in coronary artery disease (PRECAD) study (study registration ID: DRKS00020960) the serum proteome of 1400 patients with suspected coronary artery disease was analyzed at the German Heart Centre Munich, Technical University Munich, Germany. The patients were recruited consecutively before undergoing angiography in 2005 and 2006 and in depth phenotype data is available. Based on propensity matching based primarily on age and complexity of coronary artery disease as measured by the GENSINI score, 55 individuals were selected based on age and sex to match the transplanted subject (n=1, four technical replicates) of the current study, and the serum proteome were analyzed. Serum samples were prepared in 96-well format on the Agilent Bravo liquid handling system using a modified protocol based on the previously published plasma proteome-profiling pipeline [5,6]. Condensed, 45 µl of 100 mM Tris (pH 8.0) was added to 5 µl of blood serum for a tenfold dilution (dilution plate) and the solution was mixed thoroughly. The dilution plate was shortly centrifuged up to 500xg and 10 µl of diluted plasma were transferred to a new 96-well plate filled with 10 µl of reduction/alkylation buffer (20 mM tris(2-carboxyethyl) phosphine (TCEP), 80 mM chloroacetic acid (CAA)) per well (digestion plate). The digestion plate was shortly centrifuged up to 500xg again and heated to 99 °C for 10 min, followed by cooling to room temperature for 2 min in a PCR cycler. For digestion of the denatured proteins, 20 µl of a freshly prepared digestion mix (0.025 µg/µl trypsin and LysC in ddH2O) was added for a final digestion volume of 40 µl, followed by incubation at 37 °C, shaking (1000 rpm) over night. Following overnight digestion, the enzymatic activity was quenched by adding 60 µl of 0.2% trifluoroacetic acid (TFA). For mass spectrometry (MS) measurement, 500 ng (1 µl) of the digested peptides was loaded onto disposable Evotip C18 trap columns (Evosep Biosystems) according to the manufacturer’s instructions. Shortly,

Evotips were soaked in 1-propanol and then activated with 0.1% formic acid (FA) in acetonitrile (ACN), centrifuging at 500xg for 2 min. This was followed by renewed soaking in 1-propanol and a washing step using 0.1% FA in water. To ensure proper sample loading, 100 µl of 0.2% TFA was added to the Evotips and 1 µl of sample was added into the solution before centrifugation at 500xg for 3 min. Evotips were then washed with 0.1% FA before adding 150 µl 0.1% FA to prevent drying of the C18 material. Evotips were kept at 4 °C till measurement and digested samples were stored at -20 °C.

High-pH reserve phase fractionation. To improve protein identification for data-independent acquisition (DIA) measurements of the serum samples, a project-specific spectral library was generated. For this, 24 high-pH reverse phase fractionated fractions were collected using a spider fractionator (Preomics) [7]. The fractions were dried using a SpeedVac concentrator (Thermo Fisher Scientific) and subsequently resuspended in 0.1% FA. Peptide concentrations were measured optically at 280 nm using a NanoDrop 2000 spectrophotometer (Thermo Fisher Scientific) and 250 ng per fraction were loaded onto Evotips as described above.

Liquid chromatography–mass spectrometry/mass spectrometry (LC–MS/MS) analysis. Serum samples single shots as well as the fractions for spectral library generation, were analyzed using an Evosep One liquid chromatography (LC) system (Evosep Biosystems) coupled to an Orbitrap Exploris 480 mass spectrometer (Thermo Fisher Scientific). Peptides were eluted from the Evotips with up to 35% ACN and separated on an in-house packed 15 cm column (inner diameter of 150 µm, packed with 1.9 µm Reprosil-Pur C18 beads) using the Evosep 30 samples per day (SPD) method (44 min). The column temperature was maintained at 50 °C using an integrated sonation column oven (Sonation) and the column was interfaced with a NanoSpray Flex Ion Source (Thermo Fisher Scientific) at 2200 V. Library data was acquired using a data-dependent acquisition (DDA) top15 method. Full MS scans from 350-1400 m/z

were acquired at a resolution of 60,000 at m/z of 200 with a normalized automatic gain control (AGC) target of 300% and an injection time of 25 ms. MS/MS resolution was set to 15,000 (m/z 200) with a normalized AGC target of 200% and an injection time of 22 ms. Only precursors with charge states between 2+ to 6+ were selected for sequencing and the precursor isolation windows were set to 1.3 Thomson (Th). Previously target precursors were excluded from (re-)sequencing for 30 s. The normalized collision energy for higher-energy collisional dissociation (HCD) fragmentation was set to 30%.

Serum single shots were acquired in data-independent acquisition (DIA) mode. Full MS scans from 350-1400 m/z were acquired at a resolution of 120,000 at m/z of 200 with a normalized AGC target of 300% and an injection time of 45 ms. For MS/MS scans, the collision energy was set to 30%, the resolution to 15,000 at m/z 200, the normalized AGC target to 1000% (Tune Version 1) and the injection time to 22 ms. 49 equidistant DIA windows of 13.7 m/z with 1 m/z overlap were distributed across the m/z range of 361-1033.

MS data analysis. The DDA raw files for the generation of a deep spectral library were searched against the human reference proteome database (Uniprot, November 2016) in MaxQuant (V. 1.6.7.0) using default settings. FDR was set to 1% at both the peptide precursor and protein levels. The search output (msms.txt) file was then used to generate the library in Spectronaut (V. 13.15.200430) using default parameters and the same human reference proteome as before.

Single-shot serum proteome samples were analyzed with Spectronaut (V.14.8.201029) using default parameters. For protein level label-free quantification, the Spectronaut internal QUANT 2.0 algorithm was used. FDR was set to 1% at both the peptide precursor and protein levels.

Data preprocessing. Protein intensities were log_2 transformed for downstream analysis and the proteome dataset was filtered for 80% valid values across all samples (proteins with >20% missing values were excluded from downstream statistical analysis). In total 735 proteins were quantified in the serum proteomes and filtering for 80% valid values across all samples resulted in a dataset of 317 proteins with a data completeness of >95%. Remaining missing values were imputed by drawing random samples from a left-shifted normal distribution (probabilistic minimum). The mean was downshifted by 1.8 s.d. and scaled by 0.2 s.d. relative to that of the overall protein abundance distribution in a sample. Potential plate-wise batch effects were corrected using pyCOMBAT. Assessed on 154 quality control samples, median workflow coefficient of variation was 21.25% across a 13-week measurement period.

Statistics

Selection of the genes and proteins based on their expression/abundances. All transcriptomic and proteomic datasets were log_2 transformed. Homogeneity of the samples were assessed by PCA (Carey 1975). The R statistical programming language (v.4.0.4) was used for data analysis (R-Team 2018). PCA was performed using *'prcomp'* function included in the 'stats' package. The *'Heatmap.2', 'boxplot', 'beanplot', 'gplot',* and *'ggplot2'* libraries/packages were used for data visualization. In order to identify the most abundant genes and protein from RNA-seq and proteomics data of the patient (n=1), we used a statistical approach based on the mean and standard deviation (SD) of the expression values. By calculating the mean expression value of all the genes or proteins, and then selecting those whose expression levels fall above (mean ± 2 SD). By applying this strategy, we identified 226 and 288 abundant genes in the PBMCs and blood samples respectively, and 12 abundant proteins in the blood sample obtained from the patient.

Extreme genes or proteins are likely to have a large effect-size and may represent key biological processes or pathways that are perturbed in the individual. The strategy of selecting extreme genes or proteins based on the log-normal distribution of data can be a reliable strategy provided the data is adjusted for technical variations by using multiple replicates. Of note, the transcriptomics datasets were adjusted for measurements from two lanes data generated from the sequencers. The proteomics dataset was averaged from four technical replicates. Moreover, when dealing with a single subject, the chances of finding highly abundant genes or proteins are inherently more robust than when analyzing a larger cohort. This is because there is less inter-subject variability or heterogeneity within the data, allowing for more precise measurements and a higher signal-to-noise ratio. However, it is important to note that the results obtained from a single subject should be validated in independent datasets or through functional experiments to ensure their reliability and generalizability.

Co-expression analysis and functional modules. Co-expression analysis is a common method used to identify functionally related genes. Co-expression analysis was performed using GeneMANIA, a widely used web-based platform [8]. Gene symbols or accession numbers of the most abundant genes and proteins of interest were provided as an input to GeneMANIA. A co-expression network based on the co-expressed genes was inferred. The network depicts relationships among the selected genes, and their co-expressed partners. The co-expression network with the highly connected genes (hubs) and functional modules was selected.

Fold enrichments of the gene ontology (GO) terms. Fold enrichment analysis is a common technique used to identify enriched biological functions or pathways associated with a set of genes of interest. To perform a fold enrichment analysis using g:Profiler, the list of abundant

genes or proteins of interest was uploaded, and human reference genome was selected as a background set. g:Profiler then calculates the enrichment of GO terms in the input gene set compared to the background set using a hypergeometric test [9]. The results of the analysis are presented as a horizontal bar plots of enriched GO terms with their associated fold enrichment.

References

1. Andrews S. FastQC: a quality control tool for high throughput sequence data. http://www.bioinformatics.babraham.ac.uk/projects/fastqc

2. Bolger AM, Lohse M, Usadel B. (2014) Trimmomatic: a flexible trimmer for Illumina sequence data. *Bioinformatics* **30**:2114-2120. doi: 10.1093/bioinformatics/btu170

3. Dobin A, Davis CA, Schlesinger F*, et al.* (2013) STAR: ultrafast universal RNA-seq aligner. *Bioinformatics* **29**:15-21. doi: 10.1093/bioinformatics/bts635

4. Love MI, Huber W, Anders S. (2014) Moderated estimation of fold change and dispersion for RNA-seq data with DESeq2. *Genome Biol* **15**:550. doi: 10.1186/s13059-014-0550-8

5. Strauss MT, Bludau I, Zeng W-F*, et al.* (2021) AlphaPept, a modern and open framework for MS-based proteomics. *bioRxiv* 2021.2007.2023.453379. doi: 10.1101/2021.07.23.453379

6. Geyer PE, Kulak NA, Pichler G*, et al.* (2016) Plasma Proteome Profiling to Assess Human Health and Disease. *Cell Syst* **2**:185-195. doi: 10.1016/j.cels.2016.02.015

7. Kulak NA, Geyer PE, Mann M. (2017) Loss-less Nano-fractionator for High Sensitivity, High Coverage Proteomics. *Mol Cell Proteomics* **16**:694-705. doi: 10.1074/mcp.O116.065136

8. Warde-Farley D, Donaldson SL, Comes O*, et al.* (2010) The GeneMANIA prediction server: biological network integration for gene prioritization and predicting gene function. *Nucleic Acids Res* **38**:W214-220. doi: 10.1093/nar/gkq537

9. Raudvere U, Kolberg L, Kuzmin I*, et al.* (2019) g:Profiler: a web server for functional enrichment analysis and conversions of gene lists (2019 update). *Nucleic Acids Res* **47**:W191-W198. doi: 10.1093/nar/gkz369
